# Surgical correction of unsuccessful derotational humeral osteotomy in obstetric brachial plexus palsy: Evidence of the significance of scapular deformity in the pathophysiology of the medial rotation contracture

**DOI:** 10.1186/1749-7221-1-9

**Published:** 2006-12-27

**Authors:** Rahul K Nath, Sonya E Melcher, Melia Paizi

**Affiliations:** 1 Texas Nerve and Paralysis Institute, 2201 W. Holcombe Blvd., Houston, TX, USA

## Abstract

**Background:**

The current method of treatment for persistent internal rotation due to the medial rotation contracture in patients with obstetric brachial plexus injury is humeral derotational osteotomy. While this procedure places the arm in a more functional position, it does not attend to the abnormal glenohumeral joint. Poor positioning of the humeral head secondary to elevation and rotation of the scapula and elongated acromion impingement causes functional limitations which are not addressed by derotation of the humerus. Progressive dislocation, caused by the abnormal positioning and shape of the scapula and clavicle, needs to be treated more directly.

**Methods:**

Four patients with Scapular Hypoplasia, Elevation And Rotation (SHEAR) deformity who had undergone unsuccessful humeral osteotomies to treat internal rotation underwent acromion and clavicular osteotomy, ostectomy of the superomedial border of the scapula and posterior capsulorrhaphy in order to relieve the torsion developed in the acromio-clavicular triangle by persistent asymmetric muscle action and medial rotation contracture.

**Results:**

Clinical examination shows significant improvement in the functional movement possible for these four children as assessed by the modified Mallet scoring, definitely improving on what was achieved by humeral osteotomy.

**Conclusion:**

These results reveal the importance of recognizing the presence of scapular hypoplasia, elevation and rotation deformity before deciding on a treatment plan. The Triangle Tilt procedure aims to relieve the forces acting on the shoulder joint and improve the situation of the humeral head in the glenoid. Improvement in glenohumeral positioning should allow for better functional movements of the shoulder, which was seen in all four patients. These dramatic improvements were only possible once the glenohumeral deformity was directly addressed surgically.

## Background

Obstetric brachial plexus injury (OBPI) has been described as a discrete entity since 1754 [1]. The pathophysiology of the secondary deformities encountered in this population was described succinctly in 1905 by Whitman who wrote that the large majority of internal rotation and subluxation deformities of the shoulder in children with obstetric brachial plexus injuries were caused by fibrosis and contractures developed as a consequence of the neurological injury [2]. The medial rotation contracture (MRC) is the most significant secondary shoulder deformity in children with severe OBPI, requiring surgery in more than one third of patients whose injury did not resolve spontaneously [3].

The current surgical approach to treating persistent MRC in OBPI patients is derotational humeral osteotomy [4-12] or anterior capsule release [13]. Humeral osteotomy attempts to improve the patient's passive range of external rotation, but ignores the bone deformity at the root of persistent MRC, and does nothing to address the attendant subluxation of the humeral head within the glenoid fossa. Anterior capsule release may result in excessive external rotation positioning of the humerus with attendant loss of internal rotation and midline functioning [13].

**Figure 1:**
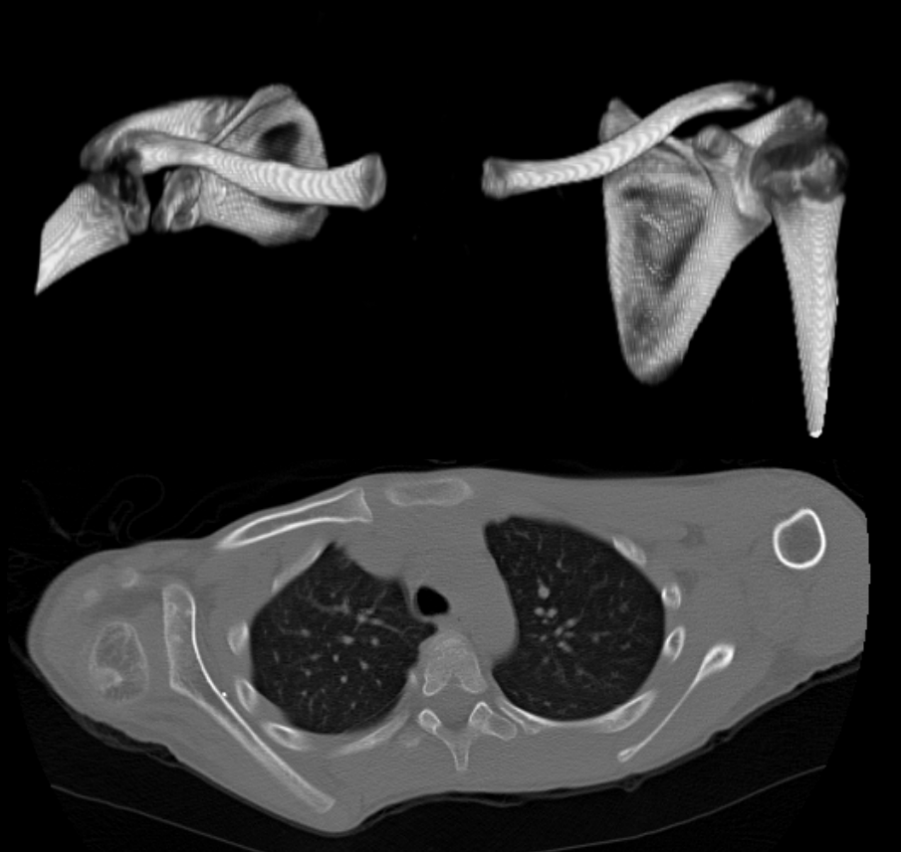
**CT images showing SHEAR deformity present after humeral osteotomy**. Ten year old boy after unsuccessful humeral osteotomy with right-sided SHEAR deformity demonstrated in 3D CT anterior view (above) and posterior subluxation demonstrated in axial view (below).

Scapular hypoplasia, elevation and rotation (SHEAR) deformity [14] is the ultimate bony manifestation of the muscular fibrosis described by Whitman, and is present in the majority of OBPI patients exhibiting MRC. The SHEAR deformity must be accounted for in any surgical correction of the MRC, and humeral osteotomy as a strategy for bony correction does not do so (Figure 1).

The presentation of weakness of the deltoid and external shoulder rotators caused by the common C5 injury seen in OBPI immediately affects growth of both the muscles and bones. Formation of contractures and consequent asymmetric muscle action on the developing bony elements of the shoulder results in bone deformation of the scapula and humerus. The scapula not only elevates and rotates laterally, but also becomes hypoplastic with flattening of the glenoid fossa and hooking of the acromion process. The clavicle and acromion process impinge upon the humeral head due to the abnormal positioning of the scapula and associated acromio-clavicular triangle (ACT), with its sides defined by the clavicular shaft and the acromion process and its base by an imaginary line connecting their medial ends. Functionally debilitating effects include medial rotation and posterior and inferior subluxation of the humerus within the glenoid fossa.

The abnormal migration of the scapula disrupts the normal anatomic relationships of the humeral head, the glenoid fossa and the acromio-clavicular triangle. Impingement of the distal acromio-clavicular triangle against the humeral head limits external rotation of the arm and shoulder. Without addressing the joint derangement, procedures such as humeral osteotomy are likely to fail or have significant rates of recurrence. To our knowledge there is no published method for correcting recurrence of the medial rotation contracture other than repeated humeral osteotomy.

A novel osseous procedure, named the "Triangle Tilt," releases and tilts the acromio-clavicular plane back to neutral thus relieving the impingement of the acromio-clavicular triangle on the humeral head. The humeral head may now reposition passively into the neutral position within the glenoid fossa. Here we report the use of this technique to treat 4 children who had undergone unsuccessful humeral osteotomies.

## Methods

During a 10 month period between October 2005 and August 2006, 73 obstetric brachial plexus patients with persistent internal rotation underwent Triangle Tilt surgery. Four of these patients had undergone previous humeral osteotomy (performed by board-certified pediatric orthopedic surgeons) with complete failure of the procedure. All 4 had residual MRC with SHEAR deformity, and underwent Triangle Tilt surgery as a salvage procedure for unsuccessful humeral osteotomy.

The presence of SHEAR deformity was determined by physical examination and confirmed by 3D-CT (computed tomography) if possible [14]. Elevation of the scapula was estimated clinically. Scapular elevation, defined as the percentage of scapula visible above the clavicle and caused by downward and anterior rotation, was quantitated on a 3D-reconstruction of the CT and confirmed the severity of the underlying SHEAR deformity.

Version and subluxation were measured on axial CT or MRI images. A scapular line was drawn connecting the medial margin of the scapula to the middle of the glenoid fossa on transverse CT or MRI (magnetic resonance imaging) images at the mid-glenoid level. The glenoscapular angle between the scapular line and a line connecting the base of the anterior labrum and posterior labrum was measured according to Friedman et al. [15]. 90° were subtracted from the posteromedial quadrant angle to determine version. The degree of humeral head subluxation was determined using the same scapular line and a perpendicular line traversing the humeral head at its greatest diameter. The distance of the scapular line to the anterior portion of the head and the greatest diameter of the humeral head were measured. The ratio of these distances multiplied by 100 determines percent subluxation.

Two of the patients were girls, ages 7.9 and 10.4 years, and 2 were boys, ages 10.4 and 11.9 years at the time of surgery. Two patients had undergone nerve surgery in infancy. Prior to humeral osteotomy, all 4 had undergone muscle contracture release, tendon transfers, and decompression of the axillary nerve at the quandrangular space [16-19]. Improvements in abduction from muscle surgery were maintained at the time of surgery. The medial rotation posture at rest was unaddressed by humeral osteotomy and was not responsive to additional therapy and splinting.

Shoulder movements were assessed preoperatively and postoperatively by evaluating video recordings of standardized movements according to the modified Mallet classification [20]. Additional measurements were made of the angle of the humerus to the trunk during the hand-to-mouth movement (trumpeter sign) and the angle of forearm supination as a more sensitive determination of functional ability. All assessments were made independently of the surgeon and principal author.

### Surgical Procedure

The Triangle Tilt surgery consisted most importantly of four components. First, osteotomy separated the clavicle at the junction of the middle and distal thirds. Second, osteotomy of the acromion process at its junction with the spine of the scapula was performed. Then, thirdly, ostectomy of the superomedial angle of the scapula was enacted. Finally, the extremity was splinted in adduction, 5° of external rotation and full forearm supination (90°). Splinting was maintained for 6 weeks after which time the splint was worn only at night for an additional 3 months.

Minor elements of the procedure included bone grafting of the acromion process osteotomy site, and semi-rigid fixation of the clavicular osteotomy segments to prevent nonunion. Since all four of these children had proven shoulder instability, particularly subluxation, diagnosed by CT or positional MRI imaging, posterior glenohumeral capsulorrhaphy was performed.

The same surgeon performed all surgical procedures (RKN).

## Results

**Figure 2:**
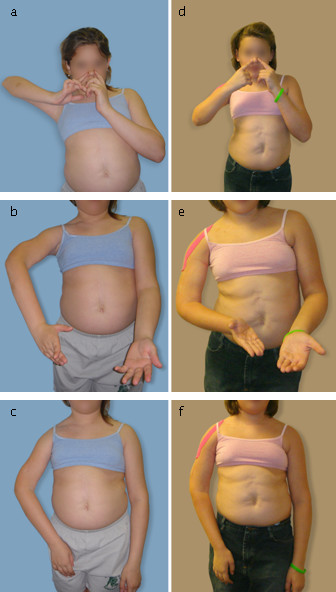
**Functional Improvement with Triangle Tilt surgery**. Pictures of 10 year old girl who had previously undergone an unsuccessful humeral osteotomy, pre (a through c) and 6 months post (d through f) Triangle Tilt surgery. Panels a and d show decreased trumpet sign during the hand to mouth movement. Panels b and e show improved supination. Panels c and f show the improvement in resting arm position.

The preoperative and postoperative Mallet scores for these patients are presented in Table 1 with representative photographs in Figure 2. The follow-up periods were 4 to 14 months with two of the four patients still undergoing nighttime splinting. There were, however, clear improvements in shoulder function which were not previously achieved with humeral osteotomy. Mallet score before Triangle Tilt surgery was 10, 16, 12 and 13. After surgery, these patients improved to 17, 19, 18, and 19, respectively. All four children were able to supinate to 60° or greater and were able to bring their hands to their mouths with a trumpeter sign of less than 45° postoperatively. Before surgery, no child was able to supinate to greater than 30° and the smallest trumpeter sign angle was 70°. Forearm supination increased secondarily to improved external rotation at the shoulder, and provided a convenient indicator of changes in external rotation. Improvements were also noticeable in the manner in which the arm was held at rest (Figure 2C and 2F).

**Table 1:** Radiographic classifications of glenohumeral deformity

	Preoperative values													Postoperative values								
Patient no.	Subluxation	Version	% Scapula visible over clavicle	Glenohumeral deformity*	Age at surgery	Abduction	External rotation	Hand to Neck	Hand to Spine	Hand to Mouth	Hand to Mouth angle	Supination angle	Total Mallet	Abduction	External rotation	Hand to Neck	Hand to Spine	Hand to Mouth	Hand to Mouth angle	Supination angle	Total Mallet	Follow-up (months)

1	13.5	-27	N/A	III	10.4	4	1	2	2	1	120	-90	**10**	4	3	3	3	4	40	60	**17**	6
2	22.2	-24	25	III	11.9	4	3	3	3	3	70	30	**16**	4	4	4	3	4	10	80	**19**	9
3	45.7	-28	N/A	III	7.9	4	1	3	2	2	110	-90	**12**	4	3	3	2	4	20	35	**16**	14
4	59.7	-42	41	V	10.4	4	2	4	2	1	135	0	**13**	4	4	4	3	4	10	90	**19**	4

Mallet scores and functional hand to mouth and forearm supination angles in patients who following failed humeral osteotomy recently underwent Triangle Tilt surgery. *Glenohumeral deformity classification according to Waters [21]. N/A data not available.

## Discussion

The developmental consequences of an obstetric brachial plexus injury, medial rotation contracture and progressive posterior dislocation of the shoulder, have serious consequences for shoulder function. Most commonly, the treatment method is humeral osteotomy, which places the arm in a more functional, externally rotated position. Though this procedure can give functional improvement, a significant proportion of children are not helped by this salvage procedure due to the fact that it does not address the bone deformities at the root of the progressive posterior dislocation and poor shoulder movement. The presence of unaddressed SHEAR deformity guarantees the continued impingement of the acromion upon the humeral head which can lead to recurrence of the debilitating internal rotation. Only in the absence of significant SHEAR is humeral osteotomy a viable treatment option.

The improvements possible with the Triangle Tilt surgery are clear from the preoperative and postoperative photographs shown in Figure 2. Mallet functional scores quantitatively show the improvements of all four patients who had previous humeral osteotomies (Table 1). One patient improved Mallet score by 3, another by 7 and the remaining two by 6 points. Satisfactory changes in function are reflected in the measured angles of forearm supination (improvement by 150, 50, 165 and 90 degrees respectively) and flaring of the elbow during the hand to mouth movement (80, 60, 35 and 45 degrees). Because of the apparent pronation deformity due to MRC pre-surgically the neutral position was inaccessible and so supination increased by more than 90° in three out of four patients.

The degree of torsion caused by contractures around the shoulder is manifest during surgery, and observation of how the bones respond during surgery reveals the forces still acting on the glenohumeral joint after humeral osteotomy. When released by Triangle Tilt, the highly abnormal bony framework around the injured shoulder and the significant intraosseous torque results in immediate clavicular and acromial movements. Separation of the distal acromio-clavicular triangle from the abnormal medial structures relieves the torsion developed over time.

The clavicle is abnormally twisted due to scapular migration, and the distal and proximal clavicle segments are intraoperatively observed to rapidly unwind after osteotomy. Significant movement also follows osteotomy of the acromion process, with the body of the acromion process and the medial margin of the acromion rapidly separating, and the distal segment moving both inferiorly and posteriorly. The distal acromio-clavicular triangle becomes normalized, and so does the humeral head through its relationship to the lateral structures. With the release of the abnormal torque and the leveling of the acromio-clavicular triangle, the glenohumeral axis returns towards neutral. This improves clinical arm positioning and movement possibilities.

## Conclusion

The four patients presented here demonstrate how important it is to recognize and treat the bone deformity. If SHEAR is present, it must be accounted for in the surgical plan. The design of the Triangle Tilt procedure aims at improving the position of the humeral head in the glenoid fossa by eliminating the impingement occurring in the SHEAR deformity. Long-term improved function of the shoulder is the expected consequence of improved glenohumeral anatomy. Only months after surgery, these four patients who had Triangle Tilt surgery to address the SHEAR as well as the medial rotation contracture show dramatically improved function.

## Competing interests

The authors declare that they have no competing interests.

## Authors' contributions

RKN conceived of the study, performed all surgeries, and edited the manuscript. MP collected and analysed data, created figures, and edited the manuscript. SEM collected and analysed data, and drafted the manuscript.
